# Equipping Nurses for Migrant Mental Health Care: An Integrative Review

**DOI:** 10.1177/10436596251372946

**Published:** 2025-09-11

**Authors:** Geneveave Barbo, Don M. Leidl, Pammla Petrucka

**Affiliations:** 1 University of Saskatchewan, Regina, Canada; 2 University of New Brunswick, Moncton, Canada

**Keywords:** cultural competency, education, nursing, emigrants and immigrants, health services accessibility, mental health services, refugees and asylum seekers, transients and migrants

## Abstract

**Introduction:**

Nurses play a pivotal role in delivering mental health care to migrants, but many lack the knowledge and training needed to effectively meet these populations’ unique needs. This integrative review examined the existing literature on best practices for caring for migrants with mental health challenges.

**Methods:**

Comprehensive searches were conducted across four databases as well as gray literature. After all eligible articles had been identified, data extraction and thematic analysis were performed.

**Results:**

A total of 54 articles were examined, revealing four major themes: (a) core frameworks and principles; (b) building trust and fostering therapeutic relationships; (c) communication strategies; and (d) assessment and treatment planning.

**Discussion:**

The findings of this review may assist health care providers, especially nurses, who are working with migrants with mental health difficulties to overcome stigma, discrimination, and cultural and linguistic barriers, thereby enhancing their health outcomes and overall health.

## Introduction

Migrant populations often face significant barriers to accessing mental health services, including stigma, discrimination, and cultural and language differences (Barbo & Petrucka, 2024; Rousseau & Frounfelker, 2019). These barriers not only impede their ability to seek and receive timely and appropriate care but also worsen their mental health conditions and overall health outcomes (Barbo & Petrucka, 2024; Boukpessi et al., 2021; Rousseau & Frounfelker, 2019; Salami et al., 2019). Nurses play a crucial role in delivering mental health care to migrants and are therefore well-positioned to address these access issues. However, many report lacking the knowledge and training needed to effectively meet these populations’ unique needs (Donlan, 2018). Major Canadian nursing associations emphasize that mental health nurses must not only understand how age, gender identity, culture, ethnicity, language, stigma, and social exclusion affect their clients’ health outcomes but also actively engage in combating stereotypes, discriminatory behaviors, and promoting inclusivity (Canadian Association of Schools of Nursing, 2015; Canadian Federation of Mental Health Nurses, 2014; Canadian Nurses Association, 2019; Registered Psychiatric Nurse Regulators of Canada, 2014). This emphasizes the urgent need for enhanced mental health education for nurses that equip them with the skills necessary to effectively address and overcome the access barriers faced by migrants with mental health challenges.

Despite the critical importance of these issues, there is a scarcity of literature offering guidance to nurses on how to competently care for these populations (Clark et al., 2010; Etowa, 2020; Willey et al., 2022). Examining guidance or strategies from other health care professionals (HCPs) that can be adapted to nursing practice may be beneficial. Another significant gap is the lack of recent reviews on this topic that also consider the access barriers experienced by migrants. For instance, one of the latest reviews on this topic was conducted by Procter (2006), who focused on providing insights to nurses delivering mental health crisis intervention to asylum seekers in emergency departments. This gap in the literature leaves nurses without the necessary tools and knowledge to provide culturally competent and effective mental health care to migrants.

The purpose of this integrative review is therefore to aggregate and examine the existing literature on best practices for caring for migrants with mental health challenges, with a particular focus on the access barriers that nurses can mitigate in practice. By identifying these practices, this review will inform the later development of immersive virtual reality simulations for nurse education, however this is not the focus of this current review. This work not only seeks to fill a critical gap in the literature but also aims to enhance nursing education and practice, leading to better health outcomes for migrant populations.

## Methods

The framework for conducting an integrative review by Coughlan et al. (2013), Toronto and Remington (2020), and Whittemore and Knafl (2005) were utilized. First, the key concepts were identified according to the review aim. These key concepts included “migrants,” “mental health conditions”, “health care delivery and access”, and commonly reported barriers to mental health access by migrants (i.e., stigma, discrimination, and lack of cultural competencies). An initial search using these concepts was then conducted in CINAHL and OVID MEDLINE (R) to identify the relevant search terms and articles. This was performed through the examination of the titles, abstracts, and index terms of potentially relevant articles. From this, a preliminary search strategy was developed (refer to Table 1). Next, this preliminary search strategy was used to create a comprehensive search strategy that is specific to each chosen database (i.e., CINAHL, OVID MEDLINE [R], PsycINFO, and Web of Science). The assistance of a Health Sciences librarian was sought to refine this search strategy (see Table 2).

**Table 1.** Preliminary Search Strategy.

	Key concepts	Search terms
1	Migrants	migrant* OR newcomer* OR immigrant* OR refugee* OR asylum seeker* OR forced displacement OR “refugee identity” OR “refugee background”
2	Mental health conditions	“mental health” OR “mental health disorder*” OR “mental health condition*” OR “mental health illness*” OR “anxiety disorder*” OR “mood disorder*” OR “post*traumatic stress disorder*” OR PTSD OR schizophrenia OR “psychotic disorder*” OR depress* OR anxiety
3	Healthcare delivery and access	(healthcare OR “health care”) AND (delivery OR access*) OR “access to healthcare” OR “access to health care” OR “service access” OR accessibility OR “service utili*ation” OR “utili*ation of health service*” OR “health service*” OR “health support*”
4	Commonly reported barriers to access by migrants (stigma, discrimination, and lack of cultural competencies)	stigma OR prejudice OR discriminat* OR stereotyp* OR "implicit bias” OR “cultural competenc*" OR “cultural awareness” OR "cultural sensitivity" OR “cultural humility” OR “cultural safety”

*Note.* 5 = 1 AND 2 AND 3 AND.

**Table 2.** Final Search Strategy.

CINAHL CINAHL Plus with Full Text	OVID MEDLINE (R) 1946 to September 12, 2023	APA PSYCINFO 1967 to September Week 1 2023	WEB OF SCIENCE Clarivate Analytics (Firm) issuing body
S1. migrant* S2. newcomer* S3. immigrant* S4. refugee* S5. “asylum seeker*” S6. “forced displacement” S7. “refugee identity” S8. “refugee background” S9. MH "Transients and Migrants" S10. MH "Relocation" S11. MH "Emigration and Immigration" S12. MH "Refugees"	1. migrant* 2. newcomer* 3. immigrant* 4. refugee* 5. asylum seeker* 6. forced displacement 7. refugee identity 8. refugee background 9. exp "emigrants and immigrants"/ 10. "transients and migrants"/ 11. Refugees/	1. migrant* 2. newcomer* 3. immigrant* 4. refugee* 5. asylum seeker* 6. forced displacement 7. refugee identity 8. refugee background 9. exp "emigrants and immigrants"/ 10. "transients and migrants"/ 11. Refugees/	1. migrant* 2. newcomer* 3. immigrant* 4. refugee* 5. asylum seeker* 6. forced displacement 7. refugee identity 8. refugee background
S13. S1 or S2 or S3 or S4 or S5 or S7 or S8 or S9 or S10 or S11 or S12	12. 1 or 2 or 3 or 4 or 5 or 6 or 7 or 8 or 9 or 10 or 11	12. 1 or 2 or 3 or 4 or 5 or 6 or 7 or 8 or 9 or 10 or 11	9. 1 or 2 or 3 or 4 or 5 or 6 or 7 or 8
S14. “mental health” S15. “mental health disorder*” S16. “mental health condition*” S17. “mental health illness*” S18. “anxiety disorder*” S19. “mood disorder*” S20. “post*traumatic stress disorder*” S21. PTSD S22. schizophrenia S23. “psychotic disorder*” S24. depress* S25. anxiety S26. MH "Mental Health" S27. MH "Depression+" S28. MH "Anxiety Disorders+"	13. mental health 14. mental health disorder* 15. mental health condition* 16. mental health illness* 17. anxiety disorder* 18. mood disorder* 19. post*traumatic stress disorder* 20. PTSD 21. schizophrenia 22. psychotic disorder* 23. depress* 24. anxiety 25. Mental health/ 26. exp *Anxiety disorders/ 27. exp Mood disorders/ 28. exp "Schizophrenia spectrum and other psychotic disorders"/ 29. exp "Trauma and stressor related disorders"/	13. mental health 14. mental health disorder* 15. mental health condition* 16. mental health illness* 17. anxiety disorder* 18. mood disorder* 19. post*traumatic stress disorder* 20. PTSD 21. schizophrenia 22. psychotic disorder* 23. depress* 24. anxiety 25. Mental health/ 26. exp *Anxiety disorders/ 27. exp Mood disorders/ 28. exp "Schizophrenia spectrum and other psychotic disorders"/ 29. exp "Trauma and stressor related disorders"/	10. mental health 11. mental health disorder* 12. mental health condition* 13. mental health illness* 14. anxiety disorder* 15. mood disorder* 16. post*traumatic stress disorder* 17. PTSD 18. schizophrenia 19. psychotic disorder* 20. depress* 21. anxiety
S29. S14 or S15 or S16 or S17 or S18 or S19 or S20 or S21 or S22 or S23 or S24 or S25 or S26 or S27 or S28	30. 13 or 14 or 15 or 16 or 17 or 18 or 19 or 20 or 21 or 22 or 23 or 24 or 25 or 26 or 27 or 28 or 29	30. 13 or 14 or 15 or 16 or 17 or 18 or 19 or 20 or 21 or 22 or 23 or 24 or 25 or 26 or 27 or 28 or 29	22. 10 or 11 or 12 or 13 or 14 or 15 or 16 or 17 or 18 or 19 or 20 or 21
S30. (healthcare OR “health care”) AND (delivery OR access*) S31. “access to healthcare” S32. “access to health care” S33. “service access” S34. accessibility S35. “service utili*ation” S36. “utili*ation of health service*” S37. “health service*” S38. “health support*” S39. MH "Health Care Delivery+"	31. (healthcare OR health care) AND (delivery OR access*) 32. access to healthcare 33. access to health care 34. service access 35. accessibility 36. service utili*ation 37. utili*ation of health service* 38. health service* 39. health support* 40. exp "Delivery of Health Care"/	31. (healthcare OR health care) AND (delivery OR access*) 32. access to healthcare 33. access to health care 34. service access 35. accessibility 36. service utili*ation 37. utili*ation of health service* 38. health service* 39. health support* 40. exp "Delivery of Health Care"/	23. (healthcare OR health care) AND (delivery OR access*) 24. access to healthcare 25. access to health care 26. service access 27. accessibility 28. service utili*ation 29. utili*ation of health service* 30. health service* 31. health support*
S40. S30 or S31 or S32 or S33 or S34 or S35 or S36 or S37 or S38 or S39	41. 31 or 32 or 33 or 34 or 35 or 36 or 37 or 38 or 39 or 40	41. 31 or 32 or 33 or 34 or 35 or 36 or 37 or 38 or 39 or 40	32. 23 or 24 or 25 or 26 or 27 or 28 or 29 or 30 or 31
S41. stigma S42. prejudice S43. discriminat* S44. stereotyp* S45. "implicit bias” S46. “cultural competenc*” S47. “cultural awareness” S48. “cultural sensitivity” S49. “cultural humility” S50. “cultural safety” S51. MH "Stigma" S52. MH "Prejudice+" S53. MH "Discrimination+" S54. MH "Implicit Bias" S55. MH "Stereotyping" S56. MH "Cultural Competence" S57. MH "Cultural Safety"	42. stigma 43. prejudice 44. discriminat* 45. stereotyp* 46. implicit bias 47. cultural competenc* 48. cultural awareness 49. cultural sensitivity 50. cultural humility 51. cultural safety 52. Social stigma/ 53. exp Prejudice/ 54. exp Social Discrimination/ 55. Stereotyping/ 56. Cultural Competency/	42. stigma 43. prejudice 44. discriminat* 45. stereotyp* 46. implicit bias 47. cultural competenc* 48. cultural awareness 49. cultural sensitivity 50. cultural humility 51. cultural safety 52. Social stigma/ 53. exp Prejudice/ 54. exp Social Discrimination/ 55. Stereotyping/ 56. Cultural Competency/	33. stigma 34. prejudice 35. discriminat* 36. stereotyp* 37. implicit bias 38. cultural competenc* 39. cultural awareness 40. cultural sensitivity 41. cultural humility 42. cultural safety
S58. S41 or S42 or S43 or S44 or S45 or S46 or S47 or S48 or S49 or S50 or S51 or S52 or S53 or S54 or S55 or S56 or S57	57. 42 or 43 or 44 or 45 or 46 or 47 or 48 or 49 or 50 or 51 or 52 or 53 or 54 or 55 or 56	57. 42 or 43 or 44 or 45 or 46 or 47 or 48 or 49 or 50 or 51 or 52 or 53 or 54 or 55 or 56	43. 33 or 34 or 35 or 36 or 37 or 38 or 39 or 40 or 41 or 42
S59. S13 and S29 and S40 and S58	58. 12 and 30 and 41 and 57	58. 12 and 30 and 41 and 57	44. 9 and 22 and 32 and 43

In addition to academic databases, gray literature was also sought by reviewing relevant websites, organizational platforms, conference proceedings, and dissertations and theses. Some examples of these include Mig-HealthCare project; World Health Organization; Refugee Health YYC; Center for Addiction and Mental Health; Caring for Kids New to Canada; Multicultural Mental Health Resource Center; and Mental Health Commission of Canada. Ancestry and offspring searches were also performed. Once all relevant published and gray literature were retrieved, duplicates were manually removed, and the rest were imported to Rayyan, for ease of initial screening (Ouzzani et al., 2016). At first, the title and abstracts of the articles was screened against the inclusion criteria. Then, the full-text versions of those articles previously deemed eligible were reviewed and selected/rejected for inclusion. When all eligible articles have been identified, data extraction commenced and carried out by the lead author. This data was analyzed using thematic analysis following Braun and Clarke (2006) and categorized into themes.

### Inclusion ad Exclusion Criteria

Since the ultimate purpose of this review is to gather a wide breadth of literature that will be used as the foundation for development of an immersive virtual reality simulation, empirical, secondary, anecdotal, theoretical, and gray literature were considered for inclusion. Articles targeting a broad range of HCPs were also included, recognizing that strategies from other disciplines can be relevant and adapted to nursing practice. Moreover, there were no restrictions based on publication year, but only English or French articles were selected. Also, only studies detailing strategies, approaches, interventions, and recommendations addressing mental health access barriers for immigrants, refugees, and asylum seekers, particularly those that can potentially be adopted by nurses, were included. Articles suggesting systemic or policy-level changes, requiring specialized training, or targeted interventions for migrants were excluded. In addition, studies focusing primarily on the development, validation, or assessment of simulation-based education interventions were excluded. Emphasis was placed on anti-stigma and anti-discrimination strategies as well as cultural competence and humility programs for HCPs that focus on the specific needs of migrants with mental health difficulties. Hence, articles that are aimed for the general population and those who do not have mental health difficulties were excluded.

## Results

**Figure 1.**
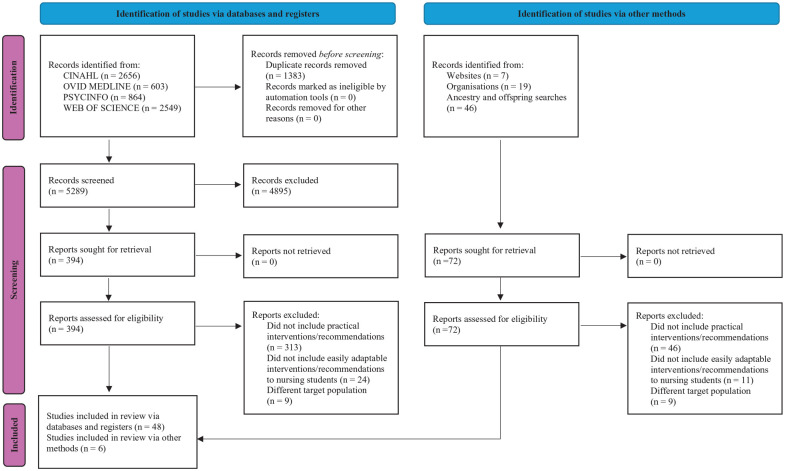
PRISMA 2020 Flow Diagram

This review included 54 articles, as detailed in the PRISMA flow diagram located in Figure 1 (Page et al., 2021). Of these, 28 are reviews, nine qualitative studies, seven book chapters, four case studies, three books, one guideline, one doctoral dissertation, and one website. The articles were predominately from the United Stated of America (25 articles), Australia (9), Canada (4) and the United Kingdom (4), as well as various European and Asian countries. Comparatively, 40 articles did not specify the target population’s country of origin. For those that did, migrants were from various countries in Africa, Asia, Central America, Europe, South America, and the Caribbean. The primary audience of these articles is mental health providers (17 articles), general health providers (15), and a smaller focus on nurses (6), psychologists, psychiatrists, counselors, and other health professionals. Additional details are available in the Supplemental Material.

From these articles, five major themes emerged: core frameworks and principles; building trust and fostering therapeutic relationships; communication strategies; and assessment practices and treatment planning (see Table 3 for the proposed curriculum framework for mental health care of migrants). Although these themes were categorized separately, it is important to note that they consist of interconnected concepts that are inseparable and mutually dependent in practice.

**Table 3.** Curriculum Framework for Mental Health Care of Migrants.

Core frameworks and principles	Building trust and fostering therapeutic relationships	Communication strategies	Assessment practices and treatment planning
• Cultural competency ○ Cultural awareness ■ Self-reflection ○ Cultural knowledge ■ Health beliefs ■ Cultural background ■ Personal identities ■ Languages ■ Social norms ■ Religious practices ■ Acculturation experience ■ Political and humanitarian context of country of origin ■ Conceptualization of mental illness ○ Cultural sensitivity	• Healthcare professionals core qualities ○ Warmth ○ Caring ○ Calming ○ Friendliness ○ Compassion ○ Empathy ○ Curiosity ○ Respectfulness ○ Humility ○ Nonjudgemental ○ Open-mindedness ○ Flexibility ○ Non-threatening, non-intrusive demeanor	• Culturally and linguistically- appropriate communication ○ Open-ended questions ○ Avoid medical jargon ○ Break down into manageable parts ○ Regularly check client’s understanding ○ Non-verbal behaviors ■ Eye contact ■ Smiling/non-smiling ■ Respect personal space ■ Avoid demeaning body language/s ■ Covey attentiveness and empathy	• Client Assessment ○ Understanding of mental illness from client’s perspective ■ Onset ■ Symptoms ■ Perceived causes ■ Beliefs about illness causes and consequences of illnesses ○ Cultural and social factors influencing perception of illness ■ Religious beliefs ■ Ethnic identity ■ Community reactions ○ Life experiences and background of client
• Cultural humility	• Establishing a safe space ○ Explain roles and process ○ Explain available resources ○ Set realistic goals ○ Inform migrants about their rights to: ■ Autonomy ■ Privacy ■ Confidentiality	• Use cultural brokers if possible	• Treatment expectations and preferences ○ Preferences for traditional or modern therapies ○ Goals for recovery
• Client-centered care		• Interpreters: avoid relatives or untrained staff workers	
• Family-centered care			
• Trauma-informed care			
• Intersectionality approach			

### Core Frameworks and Principles

Six primary frameworks for HCPs working with migrants facing mental health challenges have been identified from the reviewed articles. The subsequent section will explore each framework, while discussing their foundational principles and their application in clinical settings in detail.

#### Cultural Competency

Numerous articles emphasize the importance of HCPs’ cultural competency in effectively addressing the mental health needs of migrant populations (Ellis et al., 2020; Etowa, 2020; Ibrahim & Heuer, 2016; Mollah et al., 2018; Schouler-Ocak et al., 2015; Suurmond et al., 2010). According to Ellis et al. (2020) and Etowa (2020), cultural competency is developed first through reaching cultural awareness, followed by cultural knowledge and cultural sensitivity. Cultural awareness requires HCPs to engage in self-reflection to critically assess their own values, beliefs, biases, and cultural identities (Clark et al., 2010; Dana & Allen, 2008; Due & Currie, 2022; Etowa, 2020; Fiorillo et al., 2016; Ha Kwong, 2011; San Lau & Rodgers, 2021; Schouler-Ocak et al., 2015; Suurmond et al., 2010; Vanstone et al., 2012). This process helps them recognize and correct misconceptions and ingrained prejudices, therefore ensuring these biases do not influence their care provision ((Etowa, 2020; Qureshi et al., 2008). Without consistent self-reflection, these erroneous beliefs may remain unchallenged.

While cultural knowledge has been described as the essential information HCPs need when caring for migrants, including their health beliefs, cultural background, personal identities, languages, social norms, religious practices, acculturation experience, the political and humanitarian context of their country of origin, and conceptualization of mental health (Ellis et al., 2020; Etowa, 2020; Ha Kwong, 2011; Moleiro et al., 2013; Saherwala et al., 2021; San Lau & Rodgers, 2021; Suurmond et al., 2010; Vanstone et al., 2012). Etowa (2020) and Moleiro et al. (2013) suggest using this comprehensive knowledge to develop deeper, tailored insights for each cultural group, while meticulously analyzing the context behind the clients’ statements and their underlying thought processes.

Cultural sensitivity is developed by fostering trust, acceptance, and respect, while being attuned to the clients’ cultural backgrounds, societal contexts, and traditions (Almoshmosh et al., 2019; Corneau & Stergiopoulos, 2012; Etowa, 2020; Ha Kwong, 2011). This involves acknowledging and valuing their life experiences, beliefs, and strengths and understanding how individuals express their identities within their cultural and religious contexts (Almoshmosh et al., 2019; Corneau & Stergiopoulos, 2012; Etowa, 2020; Ha Kwong, 2011). Yet, it is crucial to remember that individuals may not fully adhere to their cultural practices for various reasons, emphasizing the importance of respecting personal choice.

#### Cultural Humility

Given the significant diversity within migrant populations, cultural humility builds on the premise that the HCP can never entirely know and master every culture and subculture (Culturally Connected, n.d.). As such, they should adopt a humble stance and acknowledge that individuals from those cultures are the true experts. This mind-set discourages assumptions or overgeneralizations, stemming from the HCPs’ education and experiences (Adams & Kivlighan, 2019; Clark et al., 2010; Due & Currie, 2022; Garcini et al., 2022; Okoro, 2022; Vanstone et al., 2012). Open discussions with clients about their beliefs, values, preferences, and needs as well as adopting a “not knowing” perspective is therefore recommended (Ibrahim & Heuer, 2016; Okoro, 2022; Vanstone et al., 2012). Embracing uncertainty enables HCPs to learn from the clients’ perspectives, while encouraging collaborative, non-paternalistic partnerships that dismantle traditional power dynamics (Culturally Connected, n.d.; Dana & Allen, 2008; Due & Currie, 2022; Ellis et al., 2020; Ibrahim & Heuer, 2016).

#### Client-Centered Care

Client-centered care shifts traditional medically-focused and problem-based models into a holistic and contextual approach that recognizes individuals as unique entities with specific background, preferences, and needs (Pavlish et al., 2010). It begins with establishing a shared understanding of problems, goals, and management plans between HCPs and clients at the outset of the health care encounter (Fiorillo et al., 2016). Throughout the treatment process, this approach then ensures that care practices, especially for migrants facing mental health challenges, are tailored according to their cultural, social, and personal experiences and preferences (Due & Currie, 2022; Pavlish et al., 2010). As the health care engagement concludes, feedback from patients is generally solicited as well as any remaining questions or concerns (Fiorillo et al., 2016).

#### Family-Centered Care

Family-centered care (FCC) places a heavy emphasis on the crucial role of the family and each of its members in the health and well-being of the clients (Cross & Bloomer, 2010; Lee & Mock, 2005). It is predicated on the idea that family involvement is vital to successful treatment as such engagement can improve medical compliance, reduce stigma, and support the clients throughout treatment (Cross & Bloomer, 2010; Hundley & Lambie, 2007; Lee & Mock, 2005). FCC is particularly effective for migrants with mental health challenges, as it aligns with the cultural norms and expectations prevalent in many cultures that emphasize family involvement and interconnectedness (Kim-Goh et al., 2015). Within such populations, FCC is demonstrated through respecting the family structure, dynamics, and values, which assist in building trust and facilitating better communication with clients and their family (Cross & Bloomer, 2010; Saherwala et al., 2021). In addition, geographical proximity of the family to the client must be considered as it significantly influences the caregiving dynamic and outcomes (Moleiro et al., 2013).

#### Trauma-Informed Care

Trauma-informed care is essential for addressing the mental health needs of migrants who have often faced severe trauma. It focuses on understanding, recognizing, and responding to the effects of all types of trauma, while avoiding re-traumatization (Babakarkhil et al., 2017; Schippert et al., 2023). This is accomplished through assessing and modifying treatment settings and policies that could trigger traumatic memories, such as lack of privacy, pressure to use psychotropic medications, or restricted access to services (Ibrahim & Heuer, 2016). Identifying specific triggers also help to normalize traumatic stress reactions as well as developing personalized coping plans that clients can apply independently (Ibrahim & Heuer, 2016). It is crucial to acknowledge and respond to clients’ behavioral and emotional reactions to triggers, as ignoring these reactions may intensify rather than diminish them (Ibrahim & Heuer, 2016). Sensitive communication about trauma is also vital, involving careful phrasing, avoiding detailed questions about trauma unless disclosed by the client, and understanding their reluctance to discuss trauma due to fear, shame, or embarrassment (Almoshmosh et al., 2019; Due & Currie, 2022; San Lau & Rodgers, 2021; Schippert et al., 2023; Vanstone et al., 2012).

#### Intersectionality Approach

The application of an intersectionality lens in mental health care allows HCPs to better understand and serve their clients by considering their varied social and cultural identities and how these intersect to affect their access to and experiences with mental health services (Ellis et al., 2020; Etowa, 2020). For instance, a Rwandan refugee's identities as Rwandan, Christian, and immigrant might evolve upon resettlement, where they might be perceived primarily through their racial identity (Ellis et al., 2020). This perception may lead HCPs to overlook challenges specific to their religion and immigrant status, such as finding welcoming support groups or churches (Ellis et al., 2020). These overlapping identities can also heighten barriers to seeking help, especially for migrants facing mental health issues (Etowa, 2020).

### Building Trust and Fostering Therapeutic Relationships

Building trust and fostering a therapeutic alliance are cornerstones in encouraging engagement and providing effective mental health care to migrants (Baker et al., 2016; Due & Currie, 2022; Qureshi et al., 2008; Suurmond et al., 2010). The capacity of HCPs to create a trust-based relationship is pivotal, not just as an initial engagement strategy, but also as an ongoing foundational element of care. Specific core qualities of HCPs and establishing safe space are facilitators to building trust and fostering therapeutic relationship with migrants with mental health difficulties.

#### HCPs’ Core Qualities

Personal qualities and behaviors that foster therapeutic relationships between HCPs and migrant clients include warmth, friendliness, compassion, empathy, curiosity, respectfulness, humility, nonjudgement, open-mindedness, and unhurried approach, non-threatening, non-intrusive demeanor, among others (Almoshmosh et al., 2019; Baker et al., 2016; Clark et al., 2010; Due & Currie, 2022; Fondacaro & Harder, 2014; Lee & Mock, 2005; Rogers-Sirin et al., 2015; Schouler-Ocak et al., 2015; Shepherd, 2022). While these characteristics are often presented as ideal attributes, their development must be considered within the context of real-world clinical environments that are frequently constrained by time pressures, systemic inequities, and emotional demands. Therefore, instead of treating this as a checklist of traits, it is more appropriate to approach these qualities as evolving capacities—ones that can be cultivated over time through reflective practice, culturally safe training, and supportive clinical placements. Even subtle elements, such as body language and attire, contribute to conveying respect and non-threatening presence (Cross & Bloomer, 2010; Garcini et al., 2022; Isakson et al., 2015; Saherwala et al., 2021). An essential consideration is the appropriateness of clothing when visiting homes; for instance, avoiding short dresses or ensuring legs are covered to respect cultural norms in certain contexts (Cross & Bloomer, 2010). The failure to adapt to these norms could lead to being disregarded, especially when attempting to provide mental health education (Cross & Bloomer, 2010).

#### Establishing Safe Space

Creating a safe, welcoming environment is crucial for HCPs treating migrants with mental health issues (Cross & Bloomer, 2010; Fondacaro & Harder, 2014). This begins by clearly explaining the roles and the mental health care process at the onset of health care interactions while using racially and gender-inclusive materials and visual cues ((Almoshmosh et al., 2019; D’souza et al., 2022; Ibrahim & Heuer, 2016; Suurmond et al., 2010). HCPs should inform patients about the services provided, their process, available assistance, and set realistic expectations, particularly for migrants unfamiliar with these services ((Alessi & Kahn, 2017; Crowley, 2009; Hundley & Lambie, 2007). Clarifying that the purpose of the appointment is distinct from any governmental psychological assessments related to immigration can prevent patients from feeling interrogated. These strategies also help establish professional boundaries and correct misconceptions, such as the belief that therapy provides an immediate cure (Kim-Goh et al., 2015; Lee & Mock, 2005; Okoro, 2022; Pavlish et al., 2010; Suurmond et al., 2010; Tribe & Farsimadan, 2022).

Furthermore, numerous authors advocate for informing migrants with mental health issues about their rights to autonomy, privacy, and confidentiality, especially those who may distrust health care systems due to negative past experiences (Alessi & Kahn, 2017; Almoshmosh et al., 2019; Babakarkhil et al., 2017; Clark et al., 2010; Crowley, 2009; Due & Currie, 2022; Hundley & Lambie, 2007; Sanchez & Gaw, 2007). Addressing potential fears of exposure to family or community is vital for fostering open communication and security (Hundley & Lambie, 2007).

### Communication Strategies

Effective communication is foundational in delivering competent mental health care to migrants, requiring not just clinical proficiency but also an understanding of culturally and linguistically appropriate communication strategies (Qureshi et al., 2008). HCPs must manage complex intercultural interactions that entail varying communication styles, non-verbal cues, and linguistic differences, as well as potential gaps in mental health terminologies (Cross & Bloomer, 2010; Qureshi et al., 2008). To facilitate communication with migrants, HCPs should use open-ended questions, avoid medical jargon, break down information into manageable parts, and regularly check the clients’ understanding (Culturally Connected, n.d.; Fiorillo et al., 2016; Pavlish et al., 2010; Shepherd, 2022). In addition, non-verbal behaviors, such as appropriate eye contact and smiling, respecting personal space, and avoiding demeaning body language or tones, are crucial in conveying attentiveness and empathy (Culturally Connected, n.d.; Davidson et al., 2004; Dominicé Dao et al., 2018; Fiorillo et al., 2016; Shepherd, 2022). Visual aids tailored to the client's cultural background, such as diagrams, videos, and pictures, and written texts may also assist in clarifying complex information (Culturally Connected, n.d.; Dominicé Dao et al., 2018; Fondacaro & Harder, 2014; Vanstone et al., 2012).

When required, HCPs must be prepared to collaboratively work with interpreters, opting for cultural brokers over linguistic interpreters to effectively bridge cultural differences (Cross & Bloomer, 2010; Due & Currie, 2022; Ellis et al., 2020; Raval, 2005). Ideally, in addition to cultural knowledge, such interpreters are also trained in mental health condition and treatment (Almoshmosh et al., 2019; Davidson et al., 2004; D’souza et al., 2022; Ellis et al., 2020; Schouler-Ocak et al., 2015; Tribe & Farsimadan, 2022; Tribe & Thompson, 2021).

The services of an untrained interpreter (e.g., bilingual relative or staff worker) should be limited to non-sensitive information. Employing interpreters with personal ties to the clients is discouraged due to confidentiality concerns and potential biases (Davidson et al., 2004; Searight & Searight, 2009; Tribe & Farsimadan, 2022). If there is no imminent harm, more detailed assessment can be delayed until a trained interpreter is available (Searight & Searight, 2009).

### Assessment Practices and Treatment Planning

When assessing the mental health of migrants, HCPs should comprehensively cover key areas: understanding of illness; cultural and social factors; life experiences and background; and treatment expectations and preferences. Evaluating the clients’ awareness and descriptions of their condition, including onset, symptoms, and perceived causes, is important as well as their beliefs about the consequences of the illness and any related fears, as these can influence treatment engagement (Babakarkhil et al., 2017; Clark et al., 2010 Culturally Connected, n.d.; Shepherd, 2022). Providers must also consider how cultural values, religious beliefs, ethnic identity, and community reactions influence the clients’ perception and management of their condition (Clark et al., 2010; Culturally Connected, n.d.; Goodridge, 2002; Ha Kwong, 2011; Mollah et al., 2018; Vanstone et al., 2012). In addition, being aware of the clients’ basic needs and current stressors is crucial for providing appropriate support and referrals (Vanstone et al., 2012; Willey et al., 2022).

Furthermore, the HCPs need to explore clients’ upbringing, family dynamics, and significant life events such as displacement or trauma as these impact their health beliefs, behaviors, and expectations from the health care system (Adams & Kivlighan, 2019; Mollah et al., 2018; Procter, 2005; Vanstone et al., 2012). Open discussions about the clients’ treatment expectations, preferences for traditional or modern therapies, and their overall goals for recovery must also ensue (Alessi & Kahn, 2017; Clark et al., 2010; Culturally Connected, n.d.; Shepherd, 2022). HCPs must recognize and respect the plurality of perspectives and understand that collaboration forms the most effective route to healing (Ellis et al., 2020).

In developing a treatment plan for migrants with mental health issues, a strength-based approach was recommended to build resilience and coping strategies, while promoting feelings of happiness, belonging, self-esteem, and empowerment (Crowley, 2009; D’souza et al., 2022; Due & Currie, 2022; Garcini et al., 2022; Ibrahim & Heuer, 2016). Other coping strategies that can be encouraged include assisting in the identification and/or development of social support system, stress management, relaxation techniques, establishing priorities, financial planning, informing about their legal rights, and building family connections from afar (Almoshmosh et al., 2019; Corneau & Stergiopoulos, 2012; Garcini et al., 2022).

## Discussion

This integrative review examined the literature on mental health care service delivery toward migrants to inform the development of immersive virtual reality simulations for nurses. Core frameworks and principles, building trust and fostering therapeutic relationships, communication strategies, assessment practices and treatment planning emerged as major themes. These themes highlight the complexities and particular nuances essential for addressing mental health issues among migrants. By explicitly examining these best practices, the quality of care provided may not only improve but also enhance the overall health outcomes for migrants, who often face disproportionate challenges in accessing and benefiting from mental health services.

The examination of relevant literature also revealed a significant gap in the academic and practical fields of mental health care for migrants. Despite frequent calls for more culturally tailored training for HCPs, there remains a pervasive issue of superficiality in the proposal, implementation, and evaluation of such educational programs. Several authors chose to include this recommendation; however, failure to detail the process for developing and disseminating educational materials as well as stakeholder engagement furthers the gap between the ideal and the practical applications. This superficial approach does little to advance our understanding or ability to effectively address cultural differences in health care, education, or social services. As such, individuals interested in pursuing this endeavor often find themselves needing to develop their own educational materials from scratch, similar to the aim of this review.

Furthermore, evaluations of these educational initiatives often lack sufficient detail to allow replication or a deep understanding of their effectiveness, with over 313 articles excluded from this review due to inadequate information on practical interventions. This vagueness limits the potential for building upon previous work, and the absence of detailed quantitative studies leaves a gap in actionable knowledge. Advocating for greater transparency through platforms like Cochrane, PROSPERO, and OSF.io and requiring detailed intervention descriptions could propel the field forward.

While this review represents an initial effort to consolidate literature on this topic, certain limitations must be considered. First, quality appraisal was not performed, which, although not mandatory for this review type, could have enriched the analysis. In addition, data extraction was conducted by a single reviewer, which may have introduced unintentional bias or limited the consistency of data interpretation. Employing multiple reviewers could have strengthened the reliability of the findings. Finally, the recommendations for clinical practice were also not tailored to specific cultures or sub-cultures. This oversight is noteworthy, given the diverse and unique needs of various migrant groups.

## Conclusions

Specific core frameworks and principles, building trust and fostering therapeutic relationships, communication strategies, and assessment practices and treatment planning have been demonstrated as important themes for effectively supporting migrants who face mental health issues and access barriers. While the review’s exploration unveiled a vast array of relevant literature, it also exposed certain deficiencies, notably in the depth and comprehensiveness of discussions around the development, implementation, and assessment of culturally adapted educational resources for HCPs. Moving forward, a concerted effort to increase transparency, detail, and specificity in research and practice will be crucial in bridging the gap between theoretical ideals and practical applications. Nevertheless, the insights gained from this review hold the potential to assist HCPs, especially nurses, who are working with migrants with mental health difficulties to overcome stigma, discrimination, and cultural and linguistic barriers, thereby facilitating improved mental health care for migrant populations.

## Supplemental Material

sj-docx-1-tcn-10.1177_10436596251372946 – Supplemental material for Equipping Nurses for Migrant Mental Health Care: An Integrative Review Supplemental material, sj-docx-1-tcn-10.1177_10436596251372946 for Equipping Nurses for Migrant Mental Health Care: An Integrative Review by Geneveave Barbo, Don M. Leidl and Pammla Petrucka in Journal of Transcultural Nursing
