# Supplementary material for: Equipping Nurses for Migrant Mental Health Care: An Integrative Review
Source: J Transcult Nurs. 2025 Sep 11;37(1):98–109. doi: 10.1177/10436596251372946 (PMC12722581; doi:10.1177/10436596251372946)
Supplement: sj-docx-1-tcn-10.1177_10436596251372946 – Supplemental material for Equipping Nurses for Migrant Mental Health Care: An Integrative Review [file sj-docx-1-tcn-10.1177_10436596251372946.docx]

**Supplementary Material**

**Summarized Findings**

| **Article** | **Type** | **Country of Resettlement (or authors’ country)** | **Target audience** | **Focused Population** | **Country of origin of focused population** |
| --- | --- | --- | --- | --- | --- |
| Adams & Kivlighan (2019) | Review | USA | Psychologists | Refugees | Not specified |
| Alessi & Kahn (2017) | Review | USA | Therapists | Sexual and gender minority asylum seekers | Not specified |
| Almoshmosh et al. (2019) | Review | UK, USA, Canada | Practitioners | Refugees | Syria |
| Babakarkhil (2017) | Book chapter | USA | Nurses | Refugees and asylum seekers | Not specified |
| Baker et al. (2016) | Review | Canada, Australia, UK, and US | Mental health professionals | Culturally and linguistically diverse communities, including immigrants and refugees | Burundi, Cambodia, China, Congo, Dominican Republic, Egypt, El Salvador, Greece, Haiti, India, Italy, Laos, Liberia, Mexico, Pakistan, Puerto Rico, Rwanda, Senegal, Somalia, Sudan, Togo, and Vietnam |
| Bolton et al. (2013) | Guideline | Canada | Service providers | People affected by trauma, including immigrants, refugees, and asylum seekers | Not specified |
| Clark et al. (2010) | Book chapter | USA | Nurses | Immigrants, refugees, and asylum seekers | Not specified |
| Corneau & Stergiopoulos (2012) | Review | Canada | Mental health providers | Racialized groups (including immigrants and refugees) | Not specified |
| Cross & Bloomer, (2010) | Research (Qualitative) | Australia | Mental health providers (i.e., doctors, nurses, psychologists, occupational therapists, and social workers) | Culturally and linguistically diverse populations, including immigrants | Not specified |
| Crowley (2009) | Review | USA | Nurse practitioners | Refugee children | Not specified |
| Culturally Connected (n.d.) | Grey literature | Canada | Healthcare providers | Culturally and linguistically diverse communities, including immigrants and refugees | Not specified |
| D'Souza et al. (2022) | Review | Canada, USA, Austria, the Netherlands, Germany, UK | Mental health providers | Lesbian, gay, bisexual, and transgender refugees, and asylum seekers | From those that were specified: Mexico, Nigeria, Saudi Arabia Syria, Uganda |
| Dana & Allen (2008) | Book | USA | Healthcare providers | Cultural groups, including immigrants, refugees, and asylum seekers | Not specified |
| Davidson et al. (2004) | Review | Australia | Healthcare providers | Refugee children | Not specified |
| Dominicé Dao et al. (2018) | Research (Qualitative) | Switzerland | Healthcare providers | First or second-generation migrants | Not specified |
| Dubus (2009) | Case study | USA | Healthcare professionals and paraprofessionals | Refugee | Cambodia |
| Due & Currie (2022) | Review | Australia, England, USA, and South Korea | Psychologists, psychiatrists, and other mental health practitioners | Refugee children and young people | Not specified |
| Ellis et al. (2020) | Book chapter | USA | Mental health providers | Immigrant and refugee youth | Not specified |
| Etowa (2020) | Book chapter | Canada | Nurses | Ethnocultural and racialized groups, including immigrants and refugees | Not specified |
| Fiorillo et al. (2016) | Book | Various countries | Psychiatrists | Mental health populations and specific sub-groups, including immigrants and refugees | Not specified |
| Fondacaro & Harder (2014) | Case study | USA | Graduate students in clinical psychology | Refugees | Not specified |
| Garcini et al. (2022) | Review | USA | Psychologists | Undocumented Latinx immigrants | Not specified |
| Garcini et al. (2023) | Review | USA | Mental health and medical providers, social workers, counselors, public health workers, and other allied health professionals | Undocumented status of Latinx families and youth | Not specified |
| Goodridge (2002) | Review | USA | Healthcare providers | Refugees and immigrants | Not specified |
| Ha Kwong (2011) | Research (Qualitative) | USA | Mental health providers | Racialized populations, including immigrants | Not specified |
| Hundley & Lambie (2007) | Review | USA | Mental health counselors | Russian speaking immigrants | Not specified |
| Ibrahim & Heuer (2016) | Book chapter | USA | Mental health counselors and clinicians | Ethnically diverse populations and trauma survivors, including immigrants and refugees | Not specified |
| Isakson et al., (2015) | Review | USA | Mental health providers | Trauma-exposed refugee youth and families | Not specified |
| Kim-Goh et al. (2015) | Research (Qualitative) | USA | Mental health providers | Asian Americans, including immigrants | China, Japan, Korea, Philippines, Samoa, Taiwan, and Vietnam |
| Lee & Mock (2005) | Book chapter | USA | Mental health providers | Asian families, including immigrants | China, India, Japan, Korea, Philippines, and Vietnam |
| Miller et al. (2019) | Review | USA | Primary care providers | Refugee and immigrant youth | Not specified |
| Moleiro et al. (2013) | Research (Qualitative) | Portugal | Mental health providers | Immigrants | Brazil, Cape Verde, Mozambique, New Guinea, Switzerland, and Ukraine |
| Mollah et al. (2018) | Research (Qualitative) | Australia | Mental health providers | Refugees, asylum seekers, and immigrants | Afghanistan, Greece, Italy, and Myanmar |
| Okoro (2022) | Doctoral dissertation | USA | Mental health providers | Black immigrants and refugees | Not specified |
| Pavlish et al. (2010) | Research | USA | Healthcare providers | Immigrant women | Somalia |
| Procter (2005) | Review | Australia | Nurses | Asylum seekers | Not specified |
| Procter (2006) | Review | Australia | Healthcare providers | Refugees and asylum seekers | Not specified |
| Procter (2006) | Review | Australia | Nurses | Asylum seeker | Not specified |
| Qureshi et al. (2008) | Review | Spain | Psychiatrists | Immigrants | Not specified |
| Raval (2005) | Review | UK | Mental health providers | Asylum seekers and refugees | Not specified |
| Rogers-Sirin et al. (2015) | Research (Qualitative) | USA | Mental health providers | Immigrants | Not specified |
| Saherwala et al. (2021) | Case studies | USA | Mental health providers | Muslim women, including immigrants | Not specified |
| San Lau & Rodgers (2021) | Review | Australia, Canada, England, Netherlands, Scotland, and USA | Healthcare providers | Refugees and asylum seekers | Bhutan, Bosnia, Burma, Burundi, Cambodia, Congo, Democratic Republic. Iraq, Somalia, and Sudan |
| Sanchez & Gaw (2007) | Review | USA | Healthcare providers | Filipino immigrants | Philippines |
| Schippert et al. (2023) | Review | Australia, Belgium, Canada, Denmark, New Zealand, Norway, Portugal, Sweden, UK, and USA | Healthcare providers | Torture survivors, including immigrants, refugees, and asylum seekers | Not specified |
| Schouler-Ocak et al. (2015) | Review | Germany | Mental health providers | Immigrants | Not specified |
| Searight &Searight (2009) | Review | USA | Psychologists | Immigrants and refugees | Not specified |
| Shepherd (2022) | Review | Australia | Healthcare providers | Culturally and linguistically diverse, including immigrants and refugees | Not specified |
| Suurmond et al. (2010) | Research (Qualitative) | Netherlands | Nurse practitioners | Asylum seekers | Afghanistan, Iran, Iraq, and Somalia |
| Tribe & Thompson (2022) | Review | UK | Healthcare providers | Forced migrants, including refugees and asylum seekers | Not specified |
| Tribe & Farsimadan (2022) | Review | UK | Psychologists, psychiatrists or mental health practitioners | Refugees and asylum seekers | Not specified |
| Tribe & Thompson (2021) | Book section | UK | Mental health providers | Refugees and asylum seekers | Not specified |
| Vanstone et al. (2012) | Book | Australia | Doctors, nurses and other healthcare providers | Refugees | Afghanistan, Bhutan, Burma, Burundi, China, Democratic Republic of the Congo, Ethiopia, Iran, Iraq, Liberia, Nepal, Pakistan, Sierra Leone, Somalia, Sri Lanka, Sudan, and Thailand |
| Willey et al. (2022) | Case study | Australia | Nurses | Refugees and asylum seekers | Afghanistan and Sri Lanka |
